# *PyMDA*: microcrystal data assembly using Python

**DOI:** 10.1107/S160057671901673X

**Published:** 2020-02-01

**Authors:** Lina Takemaru, Gongrui Guo, Ping Zhu, Wayne A. Hendrickson, Sean McSweeney, Qun Liu

**Affiliations:** a Biology Department, Brookhaven National Laboratory, Upton, NY 11973, USA; b Photon Sciences Division, NSLS-II, Brookhaven National Laboratory, Upton, NY 11973, USA; c Department of Biochemistry and Molecular Biophysics, Columbia University, New York, NY 10032, USA; d Department of Physiology and Cellular Biophysics, Columbia University, New York, NY 10032, USA

**Keywords:** X-ray crystallography, microcrystals, data assembly, radiation damage, multi-crystal, Python

## Abstract

The recent developments at microdiffraction X-ray beamlines are making microcrystals of macromolecules appealing subjects for routine structural analysis. Microcrystal diffraction data collected at synchrotron microdiffraction beamlines may be radiation damaged with incomplete data per microcrystal and with unit-cell variations. A multi-stage data assembly method has previously been designed for microcrystal synchrotron crystallography. Here the strategy has been implemented as a Python program for microcrystal data assembly (*PyMDA*). *PyMDA* optimizes microcrystal data quality including weak anomalous signals through iterative crystal and frame rejections. Beyond microcrystals, *PyMDA* may be applicable for assembling data sets from larger crystals for improved data quality.

## Abstract 2

A Python program to assemble radiation-damaged and partial diffraction data sets from microcrystals is described.

## 1. Introduction   

Biomolecular X-ray crystallography has enabled the understanding of biological complexity at the atomic and molecular level. The optimization of crystals to suitable sizes is a bottleneck in biomolecular crystallography. Thus, for many difficult-to-optimize samples such as membrane proteins and eukaryotic proteins, using their microcrystals for structural analysis is appealing. With the recent developments at synchrotron microdiffraction beamlines (Flot *et al.*, 2010 ▸; Evans *et al.*, 2011 ▸; Diederichs & Wang, 2017 ▸; Yamamoto *et al.*, 2017 ▸) and X-ray free-electron lasers (XFELs) (Schlichting, 2015 ▸; Spence, 2017 ▸), microcrystals produce high-resolution diffraction patterns. Compared with XFELs which produce only one diffraction pattern for every microcrystal, synchrotron microdiffraction beamlines are optimized for collection of a small wedge of rotation data from each microcrystal, thus greatly improving data quality from microcrystals (Smith *et al.*, 2012 ▸; Fuchs *et al.*, 2016 ▸; Yamamoto *et al.*, 2017 ▸). With the implementation of new data collection methods, microcrystal data acquisition at synchrotrons is now routine and maturing (Gati *et al.*, 2014 ▸; Coquelle *et al.*, 2015 ▸; Zander *et al.*, 2015 ▸; Diederichs & Wang, 2017 ▸; Owen *et al.*, 2017 ▸; Sanishvili & Fischetti, 2017 ▸; Gao *et al.*, 2018 ▸; Huang *et al.*, 2018 ▸; Basu *et al.*, 2019 ▸; Cianci *et al.*, 2019 ▸; Dauter, 2019 ▸; Guo *et al.*, 2019 ▸).

Because of radiation damage, the lifetime of a microcrystal does not allow for the collection of complete rotation data even under cryogenic conditions (Holton & Frankel, 2010 ▸; Zeldin *et al.*, 2013 ▸; Garman & Weik, 2017 ▸; Warren *et al.*, 2019 ▸). In general, only a few degrees of rotation data may be collected from a single microcrystal of a few micrometres. The problem of how to assemble data from radiation-damaged and incomplete data sets is not a trivial one. This becomes more complicated when attempting to extract weak anomalous signals for *de novo* structure determination (Liu *et al.*, 2012 ▸; Dauter, 2019 ▸). In addition, microcrystals are affected by environmental changes and their unit cells may have large variations. To rationally treat unit-cell variations, radiation damage and incomplete data in microcrystals, we and others have developed data assembly workflows (Guo *et al.*, 2018 ▸, 2019 ▸; Yamashita *et al.*, 2018 ▸; Basu *et al.*, 2019 ▸; Cianci *et al.*, 2019 ▸). With our workflow, we were able to assemble anomalous diffraction data from about 1200 native microcrystals and used the data for a successful single-wavelength anomalous diffraction analysis (Guo *et al.*, 2019 ▸).

Here we describe the implementation of our microcrystal data assembly workflow in a Python program called *PyMDA*. *PyMDA* allows for processing individual microcrystal data sets as progressive wedges to address radiation damage and allows for robust extraction of diffraction signals including weak anomalous signals through the implementation of unit-cell-based classification and an iterative outlier rejection strategy. *PyMDA* may be used routinely to process microcrystal data sets to produce one or more assembled data sets for structural analysis.

## 2. Implementation   

### 2.1. Overall workflow   

**Figure 1:**
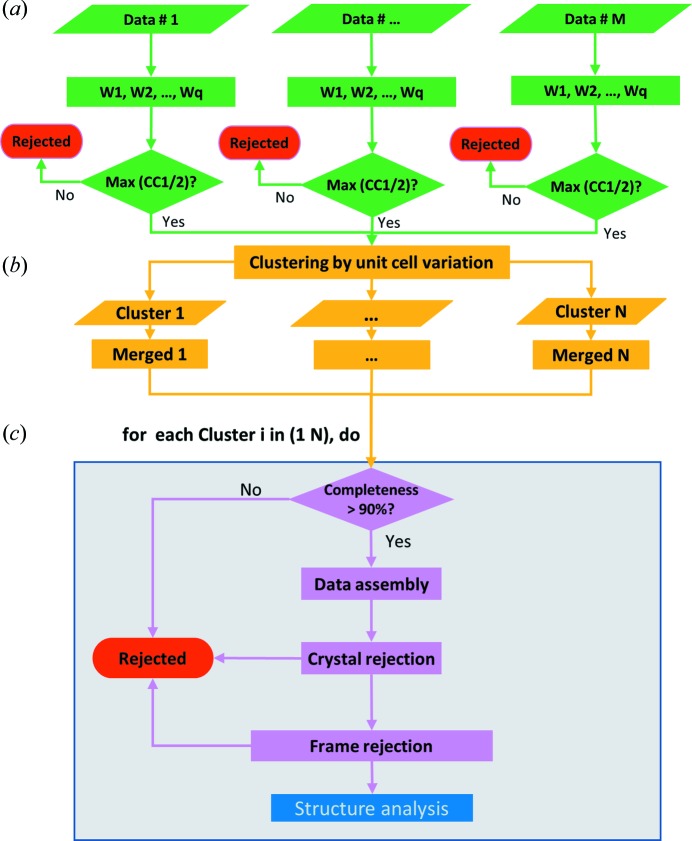
Multi-step data assembly workflow. (*a*) Progressive processing of single-crystal data sets as accumulative wedges. (*b*) Classification based on unit-cell variations. (*c*) Data assembly for each cluster that qualified (completeness > 90%). The data assembly procedure optimizes data quality by iterative crystal and frame rejections. *PyMDA* produces *N* optimized data sets, each corresponding to a different set of unit-cell parameters.

The overall workflow of our microcrystal data assembly contains three main steps and is outlined schematically in Fig. 1 ▸. The first step is to process each of *M* single-crystal data sets independently using *DIALS* (Waterman *et al.*, 2016 ▸; Winter *et al.*, 2018 ▸) and scale them using CCP4 programs *POINTLESS* and *AIMLESS* (Winn *et al.*, 2011 ▸; Evans & Murshudov, 2013 ▸) as *q* progressive wedges [Fig. 1 ▸(*a*)]. Wedge *q* includes all the preceding wedges 1 to *q* − 1. The CC_1/2_ values reported in *AIMLESS* are used for selection of those wedges. Among these *q* wedges, only the one that has the maximum CC_1/2_ is selected to form one of the *M* single-crystal data sets. The second step is to classify these *M* single-crystal data sets into *N* classes using their unit-cell parameters [Fig. 1 ▸(*b*)]. Step 3 is to assemble data sets for each of the *N* classes [Fig. 1 ▸(*c*)]. Those classes with merged data completeness greater than 90% are subject to iterative crystal and frame rejection. The quality of the assembled data is evaluated using data quality indicators: CC_1/2_ for high-resolution (diffraction limit) data and DelAnom for anomalous signals. Both CC_1/2_ and DelAnom are reported in *AIMLESS*. The assembled data sets may then be used directly for structure analysis including *de novo* phase determination.

### 2.2. Python implementation   

*PyMDA* is implemented in Python 2.7 and requires the libraries of NumPy, SciPy and Matplotlib. In addition, *PyMDA* utilizes three external programs, *DIALS*, *POINTLESS* and *AIMLESS*, for single-crystal data processing and subsequent assembly. The program *DIALS* is used for indexing and integration; *POINTLESS* is used for data combination and re-indexing if necessary; and *AIMLESS* is used for scaling and merging including iterative crystal and frame rejections. The latest versions of *DIALS*, *POINTLESS* and *AIMLESS* should be used.

*PyMDA* takes input hdf5 format data collected from modern pixel array detectors such as the EIGER 16M and 9M (Casanas *et al.*, 2016 ▸). The outputs for *PyMDA* are a series of assembled data sets that have been converted to structure-factor amplitudes with an added column for *R*free flags. *PyMDA* uses the usual CCP4 programs *CTRUNCATE*, *MTZ2VARIOUS* and *FREERFLAG* to convert intensities and to add *R*free flags.

### 2.3. Single-crystal data processing   

Because of radiation damage, each of the single-crystal data sets is typically processed as accumulative wedges using *DIALS*. *PyMDA* has a command-line option --run_dials to facilitate the use of *DIALS* for processing individual data sets. *PyMDA* runs *DIALS* tools successively from data import to data export. These tools are *dials.import*, *dials.find_spots*, *dials.index*, *dials.refine*, *dials.integrate* and *dials.export* (Waterman *et al.*, 2016 ▸). The required input parameter for --run_dials is the directory path (--hdf5_path) that contains the master hdf5 data files. Optional parameters for --run_dials are space group (--spg), unit-cell dimensions (--unit_cell), resolution (--reso), the number of wedges (--wedges) and the number of processes to be used (--thread). *PyMDA* processes many data sets automatically. However, for unknown samples, it is recommended to process a few data sets first to identify the space group and unit-cell parameters. If accurate detector parameters are known, it might be worth fixing the detector (--fix_detector). By default, *PyMDA* uses a single process, but using multiple processes is possible and preferable.

There are two resolution cutoffs used in processing single-crystal data sets. One (--reso) is the resolution at which the data will be processed, and the other one (--reso_cchalf) is for generating CC_1/2_ statistics using *AIMLESS*. The crystal is damaged, which results in a loss of intensity, particularly at high resolution. To have more frames selected for data assembly, we suggest using a statistics resolution lower than the data processing resolution (--reso) (Guo *et al.*, 2018 ▸). By default, the value of --reso_cchalf is the value of --reso plus 1.

For challenging single-crystal data sets where the diffraction is weak, we provide an optional optimization step (--opt) to optimize three spot-finding parameters, sigma_b, sigma_s and minimum spot size, used by *dials.find_spots*. With this optimization mode, *PyMDA* will run a grid search for these parameters for a maximum *I*/σ(*I*). In general, we only optimize these parameters for a selected data set; however, if desired, the optimization can be performed for all data sets. Be aware that this mode will take much longer. With known spot-finding parameters, *PyMDA* can use them directly through parameters --sigb, --sigs and --minspot.

Each single-crystal data set is processed under a different directory with the same name as the prefix of its respective hdf5 data file. After single-crystal data processing, a series of data sets in mtz format and *AIMLESS* log files are produced. These mtz files are then used for data assembly; and *AIMLESS* log files are used to extract CC_1/2_ values and unit-cell parameters. Below is an example of processing hdf5 format data:

/path-to-pymda/pymda --run_dials --hdf5_path /root-path-containing-hdf5-data/ --wedges 10 --spg p2221 --thread 4

### 2.4. Classification by unit-cell variations   

The data sets selected with the highest CC_1/2_ values are used for classification based on unit-cell variations. With the extracted *M* sets of unit-cell parameters, classification is performed (Liu *et al.*, 2012 ▸, 2013 ▸; Foadi *et al.*, 2013 ▸) with the desired number of classes. Unit-cell classification can be performed through a combination of keywords --run_mda and --ucr=N, where *N* is the number of desired classes. *PyMDA* writes out a PDF file to store the dendrogram plot that may be visualized and used as a reference to rerun the classification with a different number of classes. After unit-cell classification, *PyMDA* splits the *M* data sets into *N* classes. Below is an example of running classification for ten classes based on unit-cell variations:

/path-to-pymda/pymda --run_mda --dataprefix prefix_of_hdf5 --ucr 10

Two further classification options are provided. Option --single is to maximize the number of crystals in a single class. This option is useful to keep more crystals together for optimizing anomalous signals. The option --ward is to maximize the separation of classes and this option is useful to obtain more assembled data sets.

### 2.5. Crystal rejection   

Each of the *N* classes with data completeness higher than 90% may be used for crystal rejection with an option --rjxtal. By default, *PyMDA* produces *N* assembled data sets without crystal rejection. The goal of the crystal rejection step is to exclude single-crystal data sets that are not compatible with the merged one within each of the *N* classes. To perform crystal rejection, *PyMDA* uses the per-frame smoothed *R*_merge_ (SmRmerge) values extracted from *AIMLESS*. Prior to each iteration of crystal rejection, *PyMDA* computes the average of SmRmerge values 〈SmRmerge〉 for each crystal, sorts per-crystal 〈SmRmerge〉 and then rejects the specified number of crystals (defined by --xtal_step) with the highest 〈SmRmerge〉 values. The iteration continues until the number of crystals is equal to or less than the number defined by --xtal_step. Below is an example of running crystal rejection with a rejection of ten crystals for each iteration:

/path-to-pymda/pymda --run_mda --dataprefix prefix_of_hdf5 --ucr 10 --rjxtal --xtal_step 10

### 2.6. Diffraction frame rejection   

Following each iteration of crystal rejection, *PyMDA* can perform the rejection of those frames (--rjframe) that suffer too severely from radiation damage. Because of natural variations among microcrystals, there is no uniform rejection criterion suitable for all microcrystals. Therefore, *PyMDA* adopts a user-defined grid-search procedure for frame rejection (Guo *et al.*, 2018 ▸). Frame rejection is optional and may be performed after each iteration of crystal rejection for each of the *N* classes that qualified (*e.g.* with their data completeness higher than 90%).

By defining the sequence of decay values, frame rejection is performed under different radiation-damage conditions to optimize the overall data quality. For each single-crystal data set, *PyMDA* identifies the frame number with the lowest SmRmerge and stores it as min(SmRmerge). The frame rejection cutoff is then defined as rjframe = [min(SmRmerge) × (1 + decay)], where decay is defined as each of the values in a sequence through an option --decay. The default sequence is ‘5.0 3.0 2.0 1.0’. With the default sequence, *PyMDA* rejects frames that have rjframe values 6, 4, 3 and 2 times min(SmRmerge). Four data sets are then produced, each corresponding to a different decay value. Below is an example of running frame rejection after each iteration of crystal rejection:

/path-to-pymda/pymda --run_mda --dataprefix prefix_of_hdf5 --ucr 10 --rjxtal --xtal_steps 10 --rjframe --decay “3.0 2.0 1.0”

After crystal and frame rejection, *PyMDA* outputs multiple data sets and the associated *AIMLESS* log files, each derived from using different numbers of crystals and frames. It is suggested these data sets are sorted on the basis of different statistics reported in their *AIMLESS* log files. For a high-resolution structure analysis that does not require anomalous signals, the data set with maximum CC_1/2_ is suitable. For *de novo* structure determination that uses anomalous signals, the data set with maximum DelAnom may be used.

## 3. Discussion   

### 3.1. Microcrystal data collection and processing   

*PyMDA* assembles partial microcrystal diffraction data sets that are collected as small rotation wedges into complete crystallographic data sets. There is no special requirement for how many frames are examined by *PyMDA*. In developing *PyMDA*, we anticipate ten to 100 frames per microcrystal with a rotation angle of 0.1 to 0.3° per frame (1–30° of data per microcrystal). By processing single-crystal data sets as accumulative wedges, radiation-damaged frames should not compromise the overall data quality. Seriously damaged frames are rejected at the single-crystal data processing stage. One can use - -reso_cchalf to fine tune the control of how many frames are to be rejected from data assembly. A smaller value of - -reso_cchalf (higher resolution) results in more rejected frames.

The development of the current version of *PyMDA* was based on the use of hdf5 format data created by EIGER detectors. *PyMDA* uses the master hdf5 names to create directories to execute parallel processing. It is a requirement that all hdf5 master files are under the same root directory and have the same prefix. This is the case for data collected at NSLS-II AMX and FMX beamlines and other macromolecular crystallography beamlines using EIGER detectors. Nevertheless, these master hdf5 files can be in different locations as long as they are under the same root directory defined by --hdf5-path.

### 3.2. Resolution cutoff   

*PyMDA* provides two resolution cutoffs for single-crystal data processing and assembly. --reso is used for indexing and integration and final assembly. --reso_cchalf is used for calculation of CC_1/2_ values of single-crystal data wedges. In the single-crystal data processing stage (--run_dials), it is suggested one uses the highest resolution by visual inspection of diffraction images during or after data collection. In the assembly stage (--run_mda), different resolution cutoffs may be used. For example, if a high-resolution data set is desirable, that resolution may be used for data assembly. All crystal and frame rejections will then be optimized against that resolution. Below is an example of running frame rejection after each iteration of crystal rejection at a resolution of 2.5 Å:

/path-to-pymda/pymda --run_mda --dataprefix prefix_of_hdf5 --ucr 10 --rjxtal --xtal_step 10 --reso 2.5

If anomalous signals are to be optimized, we suggest using a lower resolution to enhance anomalous signals by increasing data redundancy. Because --run_dials and --run_mda can be performed successively, using two different resolutions through --reso can be done by running *PyMDA* twice: once at the highest resolution and once at a lower resolution (for optimized anomalous signals). Both data sets may then be used for structure refinement and phasing. For example, if the high-resolution data set is at 2.5 Å, below is an example of running *PyMDA* to optimize anomalous signals at a lower resolution of 3.5 Å:

/path-to-pymda/pymda --run_mda --dataprefix prefix_of_hdf5 --ucr 10 --rjxtal --xtal_step 10 --reso 3.5

### 3.3. Program limitations   

The current version of *PyMDA* has limitations. Firstly, *PyMDA* can only process rotation data with the hdf5 data format. Support for cbf format will be provided in a future version. Secondly, the unit-cell classification is not fully automated. We suggest checking the output dendrogram plot to define the number of classes. Because of structural changes, manipulation or poor evaluation, microcrystals might have a wide spread of unit-cell parameters. One may need to manually remove certain single-crystal data sets that have unrealistic unit cells (*e.g.* by deleting their directories) before the fully automated data assembly can be run robustly. This can be done by checking the file check.txt. This file contains the directory names, the unit-cell parameters and their corresponding CC_1/2_ values for each of the single-crystal data sets. It would be useful to manually delete these extreme data sets with quite different unit-cell dimensions. These individual data sets may also be identified by running unit-cell classification with the --single option.

## 4. Concluding remarks   

Microdiffraction data sets from microcrystals are difficult to process due to radiation damage, incompleteness and large unit-cell variations. Here we have implemented a Python program *PyMDA* for microcrystal data assembly. *PyMDA* assembles optimized data suitable for high-resolution structure analysis and *de novo* structure determination. *PyMDA* can not only assemble diffraction data from microcrystals, but also assemble data sets from larger crystals to improve data quality. *PyMDA* is available from https://github.com/qun-liu/pymda.
